# Species-specific Fungal DNA in Airborne Dust as Surrogate for Occupational Mycotoxin Exposure?

**DOI:** 10.3390/ijms9122543

**Published:** 2008-12-10

**Authors:** Anne Straumfors Halstensen

**Affiliations:** National Institute of Occupational Health, Department of Chemical and Biological Working Environment, Gydasvei 8, Pb. 8149 Dep., N-0033 Oslo, Norway E-Mail: anne.s.halstensen@stami.no; Tel. +47-23-19-53-38; Fax: +47-23-19-52-06

**Keywords:** Occupational exposure, mycotoxin exposure, inhalation, PCR, fungi

## Abstract

Possible health risks associated with occupational inhalation of mycotoxin-containing dust remain largely unknown, partly because methods for mycotoxin detection are not sensitive enough for the small dust masses obtained by personal sampling, which is needed for inhalable exposure measurements. Specific and sensitive PCR detection of fungi with mycotoxin-producing potential seem to be a good surrogate for occupational exposure measurements that include all fungal structures independent of morphology and cultivability. Results should, however, be interpreted with caution due to variable correlations with mycotoxin concentrations.

## 1. Introduction

Mycotoxins are fungal metabolites that may exert immunosuppressive, endocrine, carcinogenic and toxic effects on human and animals. Several mycotoxins are natural contaminants of grain and other agricultural products. The increasing focus on mycotoxins, particularly in the grain production industry, along with unavoidable dust exposure during crop handling, have led to a growing concern about the inhalable contribution of mycotoxin exposure in occupational settings.

The major mycotoxin classes of concern are trichothecenes, aflatoxins, fumonisins, zearalenone, and ochratoxin A, which are produced by the three fungal genera *Fusarium*, *Aspergillus* and *Penicillium* [1]. The trichothecenes comprise a large class of mycotoxins produced by several fungal genera, notably *Fusarium* species. Some of the most commonly occurring trichothecenes in grain are deoxynivalenol (DON), T-2 toxin, HT-2 toxin, nivalenol (NIV), diacetoxyscirpenol (DAS), and monoacetoxyscirpenol (MAS). Aflatoxins are primarily produced by *Aspergillus flavus* and *Aspergillus paraciticus*; fumonisins (FUM) are produced by *Fusarium verticollioides* and occur primarily in corn; zearalenone (ZEA) is produced primarily by *Fusarium graminearum*; and ochratoxin A (OTA) is primarily produced by *Penicillium verrucosum* and *Aspergillus ochraceus*. The dominant fungal species and the mycotoxins they produce may vary from one part of the world to another, depending on differences in climate and topography. At the local level, there is a high degree of mycotoxin concentration variability in crops and dust, as with their fungal producers [2–5].

The health risk from ingesting mycotoxin-contaminated agricultural products is widely acknowledged and to a certain extent controlled, but little is known whether inhalation of mycotoxin-containing dust during crop handling represents an occupational health risk. Inhaled trichothecene mycotoxins are very toxic [6–9], and may be even more toxic than dermally, orally and intraperitoneally administered mycotoxins [6, 9–10], presumably due to higher bioavailability [10–11]. Epidemiological studies have, furthermore, implicated that adverse human health effects are caused by inhalation of mycotoxins [12–14]. However, the intensity and duration of mycotoxin inhalation that cause health effects is unknown since no human effects studies of inhaled mycotoxins exist. Presently, one can therefore not determine whether adverse mycotoxin levels can be reached during different working conditions where mycotoxin-contaminated dusts are inhaled.

A proper exposure assessment is needed when evaluating health effects of work place exposure. This requires personal sampling [15] and quantitative determination of the agents of interest. The personal dust sampling equipment typically consists of a portable pump that aspirates air from the breathing zone through a sampling cassette which collects airborne dust on a filter. The sampling equipment is carried by the worker during work in order to sample dust that is representative for the workers exposure.

Mycotoxin measurements in the small dust masses obtained by personal sampling has not yet been reported, although this may in theory be possible with the low detection limits of several recent methods [5, 16–17]. Because it is easier to detect, fungi are often used as an indirect measure for mycotoxins both in agricultural and occupational settings. However, one needs to quantify and identify the fungi at the species level because the mycotoxin production depends on fungal genus, species and strain [18]. Traditional methods for fungal determination, such as microscopy and cultivation, do either not discriminate closely related species or are limited to cultivable fungi. Molecular techniques such as polymerase chain reaction (PCR) and DNA hybridization have provided significant advances in rapid identification and quantification of specific fungal DNA, irrespective of their cultivability. PCR-based detection of species-specific fungal DNA has recently been used to measure personal exposure of toxigenic *Fusarium* species [19].

This review focuses on the use of species-specific PCR to detect toxigenic fungi in personal air samples, and how this may be used to evaluate occupational mycotoxin exposure. Trichothecenes and toxigenic *Fusaria* in grain and grain dust are given special attention. The new approach prompts a thorough discussion of how to interpret the results compared to cultivation (cfu/m^3^) and microscopy (spores/m^3^).

## 2. Personal mycotoxin exposure measurements in occupational environments

Although median dust exposure in e.g. grain handling may be 5 mg/m^3^ dust [20], less than 1 mg is often collected on the filter. Analytical mycotoxin detection methods have primarily been developed to analyze food products, and are thus not optimized for the small dust masses obtained by personal sampling. This may partly explain why only few have studied occupational mycotoxin exposure [21–23].

Stationary sampling with high volume pumps is an alternative that has been used to determine airborne mycotoxin level [21–22, 24]. Other studies have used settled dust which can be obtained in larger quantities, and related the mycotoxin concentration per gram of settled dust to the level of airborne dust [5, 25]. Theoretically, grain handlers may inhale up to 34 μg mycotoxin during a workday [26].

## 3. Surrogates for mycotoxin measurements

Fungi are often used as indicators for mycotoxins both in agricultural and occupational settings, but they must be quantified and identified at the species level in order to relate the fungi to a certain mycotoxin because the mycotoxin production depends on both the fungal genus, species and strain [18]. Airborne fungi collected by impaction or filtration have primarily been identified by cultivation which limits the methods to cultivable fungi. Microscopic counting of total fungi quantifies both cultivable and non-cultivable spores, but has limited potential for identification [27–31].

### 3.1. Cultivation of fungi

Cultivable fungi may grow on semi-solid nutrient media to form colonies that can be counted with the unaided eye. Since a colony can be derived from one single microorganism or from an aggregate, the microbial exposure is expressed as colony forming units (cfu)/m^3^. Fungal colonies can be classified by their morphological appearance and eventually identified by their characteristics in culture, smell and light microscopic morphology [30]. However, rapidly growing fungi often out-compete and inhibit slowly growing species, resulting in a bias towards rapidly growing fungi [32–33]. Furthermore, various microbial species may demand different growth conditions, making optimization for each species an extensive task. Finally, colony counting may grossly underestimate the total number of microorganisms in airborne dust samples because aggregates of several individual propagules will be counted as one colony.

### 3.2. Microscopic methods

Fungi collected on filters may be directly counted in a light microscope provided they have a recognizable morphology, which unfortunately is often not the case with aerosolized microorganisms. High diversity, intra-species variability, and conflicting taxonomy of some genera, such as the *Fusarium* genus, add to this complexity. Staining of different fungal components with various fluorochromes followed by epifluorescence microscopy may facilitate microorganism recognition, although less detailed than with light microscopy [34–35]. This method is further limited by the fact that fungal spores of some species may resist staining or mask the fluorescence by dark pigmentation [36] and fungi appearing in large aggregates may lead to counting errors [34].

Scanning electron microscopy (SEM) provides a greater resolution and field depth than light and fluorescence microscopy, and allows a certain morphological recognition and classification of fungal spores and actinomycetes [37], but species identification is generally not possible. Airborne spores are subject to desiccation that may make some species, such as *Fusarium*, hard to recognize.

Although non-culture based methods may provide more valid exposure estimates than culture-based methods, their validity also depend on the ability to differentiate between species. This may be particularly important when examining fungal exposure in diseases such as allergic asthma, allergic rhinitis and hypersensitivity pneumonitis, but perhaps less obvious for “non-specific” diseases such as airway inflammation, non-allergic asthma, bronchitis and inhalation fever.

### 3.3. DNA-based fungal analysis

#### 3.3.1. Important fungal genomic DNA regions

Molecular techniques such as PCR and DNA hybridization have provided significant advances in rapid detection and characterization of specific fungal DNA, irrespective of their viability or cultivability. To utilize the technique for identification is knowledge of the fungal DNA sequence essential.

Fungal ribosomal DNA (rDNA) contains both conserved nucleotide sequences that are common to all fungi, and variable sequences that are suitable for species discrimination. The conserved fungal rRNA genes are separated by two variable internal transcribed spacer regions (ITS1 and ITS2) and organized in a tandemly repeated unit. Adjacent copies of the rDNA repeat unit are separated by an even higher variable intergenic spacer (IGS) region. Both ITS and IGS appear to evolve more rapidly than the rDNA genes, and have been used to study closely related taxa [38–39], whereas the conserved rDNA sequences have been widely used to study distantly related fungi [40].

However, high mutation rates could also cause instability of markers based on ITS and IGS. Several protein-coding genes, such as the elongation factor-1 alpha and the β-tubulin genes have therefore been explored as phylogenetic markers [39, 41].

Alternative strategies are the utilization of unique sequences in mitochondrial DNA [42] or cloned restriction fragments of genomic DNA [43]. In spite of the high polymorphism in these regions, it is not always sufficient to obtain species-specific primers, particularly when the pathogen under investigation appears together with closely related non-pathogenic species [44].

Sequence characterization of randomly amplified polymorphic DNA (RAPD) fragments reveals more sequence-specific polymorphisms than ITS-sequencing and was for the first time used by Paran and Michelmore to detect resistance genes for mildew in lettuce [45]. This method has successfully been used to discriminate between closely related *Fusarium* species such as *F. graminearum* and *F. culmorum* [46], and resulted in primers specific for *F. avenaceum* [44].

Several group-specific competitive PCR methods have quantified a number of trichothecene-producing *Fusarium* species in grain using primers based on sequences from the gene encoding trichodiene synthase (*tri5)*, which catalyses the first step in the trichothecene biosynthetic pathway [47–48]. A similar approach was used to detect aflatoxin-producing and sterigmatocystin-producing fungi [49–50], and the IGS region between *tri5* and the *tri6* gene (encoding a transcription factor) has been used to distinguish between high and low DON-producing *F.culmorum* isolates [51]. The recently characterized genes encoding various polyketide synthases required for the production of ZEA in *F. graminearum* [52–53], and OTA in *P. verrucosum* and *P. nordicum* [54], may also be used to detect fungi with specific mycotoxin-producing potential.

#### 3.3.2. Real-time PCR of toxigenic fungi in bioaerosol samples

Several PCR-based techniques may be suitable for air samples with low spore density [55–57]. However, quantitative real-time PCR using amplicon sequence non-specific fluorescent dyes [58–59] or sequence-specific fluorescent probes [60–62] is at present probably the best method for detection of airborne fungi because of the rapid, sensitive and specific quantification provided by the continuous amplification monitoring and absence of post-PCR electrophoretic needs [63–66]. Furthermore, the use of different fluorescent dyes may facilitate detection of several target microorganisms in a single reaction (multiplex PCR) [61, 66].

Quantitative real-time PCR assays have been developed to either specifically detect one particular mycotoxin-producing species, or several species with the same mycotoxin production-related genes [58, 67–68]. Several airborne fungal groups and species have been quantified by real-time PCR with the TaqMan fluorogenic hybridisation probe system [19, 69–70]. Most of these studies are based on stationary sampling, which may underestimate workers exposure to bioaerosols [71–72]. Only one study on specific fungal DNA quantification in personal samples has been published [19].

## 4. Methodological considerations

PCR has the advantage of specific identification of fungal DNA independent of cultivability, including all DNA-containing fungal structures, such as hyphae which are important contributors to mycotoxin production and bioaerosol exposure [73]. The introduction of molecular methods in occupational hygiene and indoor air has therefore improved the specificity of microbial exposure measurements and allowed rapid identification [59, 70, 74–76].

### 4.1. Detection sensitivity

The sensitivity of the PCR method is dependent on the primer sequences, and the detection sensitivity may vary 100–1000 fold for various *Fusarium* species [77]. Primers from the multiple-copy ITS sequences, may increase the sensitivity compared to primers from RAPD fragments or single copy genes such as *tri5*. A nested PCR will also increase the sensitivity compared to standard PCR [78]. Other ways to increase both the detection sensitivity and specificity is PCR followed by probe hybridization [56–57].

Detection sensitivity can be tested either by extracting DNA from a large amount of spores followed by DNA dilution or starting with a spore suspension dilution followed by DNA extraction. The first procedure gives higher detection sensitivity due to high extraction efficiency from high spore density. For samples with low spore density, which is the case for most personal air samples, the DNA extraction efficiency and recovery may be lower, and result in larger variation in detection sensitivity [56].

### 4.2. PCR inhibitors

Environmental PCR inhibiting contaminants may be co-extracted with DNA. Samples from different environments may vary in chemical and organic composition, and affect assay sensitivity differentially. Environmental compounds like phenols, humic and fulvic acids in soil, polyphosphates in fungi, heavy metals, some plant acidic polysaccharides, and high concentrations of non-target DNA may inhibit polymerase activity, thus causing false-negative results and reduced detection sensitivity [79–81]. However, PCR inhibitors may be removed by including a purification step in the extraction procedure [19, 77, 82].

Possible inhibition may be tested either by spiking the processed sample with known amount of target DNA, or spiking the unprocessed sample with known amounts of target spores. Spiking the processed sample is easiest, but it will not correct for the DNA isolation efficiency [70]. The second approach examines both the DNA isolation efficiency and the existence of any PCR inhibition substances so that the standard curve and the tested sample can be compared. However, the number of available parallel samples to be spiked for the standard curve may be limited, although the optimal solution is to spike all samples. Moreover, the standard line constructed in this procedure may not be linear due to different DNA isolation efficiency at different spore concentrations. Differences in DNA extraction efficiency may also be expected between various fungal species and between spores and hyphae. The extraction variability of common species should therefore be determined in order to standardize the extraction procedure so that all fungi in a complex sample may have similar extraction efficiencies. However, the variable microbial content in work place samples may have differential influence on the extraction efficiency and may be an unavoidable source of uncertainty associated with DNA extraction. Furthermore, spiking with target DNA may not discriminate between inhibition and no detectable target. Spiking with unrelated DNA that is not expected to be found in the samples may be more reliable as a positive internal control [68].

DNA extract dilution is known to attenuate the inhibition effect, but also to reduce the sensitivity [63, 83]. Moreover, filters of cellulose and nitrocellulose, but not polycarbonate, may inhibit PCR [84].

## 5. Microarrays

DNA microarray is another powerful tool for the parallel detection of multiple DNA sequences in one single experiment [85]. The fundamental basis for microarray is the ability of complementary DNA sequences to hybridize, but the microarray design varies depending on the research question [86–89] and several platforms exists [90].

Although the majority of microarray reports are concerned with gene expression profiling in humans, animals or plants, the use of DNA microarray technology is expanding into new fields and new applications. Several microarrays have been developed for detection of pathogens that pose threats to human, animal and plant health [81, 85, 91–92] or for better understanding of the microbial world, with particular emphasis on strain detection, assessment of microbial diversity and the structure of different communities, adaptation, expression of biologically important genes and evolution [90, 93–96]. However, microarray has thus far not been used for microbial screening of bioaerosols, which could be relevant in occupational environments.

## 6. Measurements in settled versus airborne dust

Several specific toxigenic *Fusarium* spp. have been identified and quantified in settled grain dust by species-specific semi-quantitative PCR, whereas they could not be sufficiently identified or quantified by cultivation [77]. *F. langsethia*- and tri5-specific DNA correlated fairly strong with HT-2 and T-2 (r_spearman_=0.77 and r_spearman_= 0.59, respectively, for *F. langsethiae* and r_spearman_=0.68 and r_spearman_= 0.50, respectively, for *tri5*).

Settled dust collected for mycotoxin determination may be used as surrogate for airborne dust under the assumption that settled dust is representative for airborne dust. However, as the aerosolization potential of dust components depend on microbial species, weather, agricultural equipment, and drier- and storage technology, this may not always be correct. Spatial variation in airborne dust concentration and faster sedimentation of larger particles than smaller increase the differences further.

Although *Fusarium*-DNA concentration was higher in settled dust than in airborne dust, airborne *Fusarium*-DNA was detected in personal samples even after only 10 minutes sampling time [19].

## 7. General limitations of mycotoxin surrogates

As the genes of the trichothecene biosynthetic pathway are not expressed constitutively, but are induced by developmental and environmental signals [97], the detection of potentially toxigenic fungal species may not in general predict mycotoxin presence. Presence of non-mycotoxin-producing fungi may lead to an overestimation of the predicted mycotoxin concentration. On the other hand, as the mycotoxins may be present long after the death and disintegration of the producer, an underestimation of the mycotoxin concentration is also possible. Although DNA specific for *tri5*, *F. langsethiae* and *F. poae* have been shown to correlate strongly with HT-2 and T-2 in an epidemiological study, not all expected associations were present [77]. This common problem is a limitation of the use of possible toxin-producers as indicators for toxins. Only few studies have analyzed the correlation between PCR signals and certain mycotoxin levels, and even fewer have reported positive correlations [98].

## 8. Evaluation and interpretations of data from molecular analysis

The data output from the molecular techniques are either PCR gel band intensity values or cycle threshold (C_T_) values from the real time PCR machine. The latter reflects PCR cycle number when the specific signal is detected above a certain threshold value. Although the original amount of specific DNA may be calculated for both outputs when using a known standard DNA concentration, real time PCR is more accurate. Information of DNA concentrations may be sufficient as surrogates for mycotoxins where correlations between fungi and mycotoxin have been established, but for bioaersosol exposure assessment in general, the DNA concentration should preferably be converted to a form that is applicable to occupational measures. The average ascomycetous fungal genome size is 36 Mb, corresponding to 40 fg genomic DNA. The number of cells (spores) per cubic meters of air (cells/m^3^ or spore equivalents/m^3^) has been calculated by conversion of 40 fg DNA per fungal cell (spore) [59]. Others have estimated the number of conidia detected in dust samples by using an equation that expresses the relationship between the differences in real time PCR C_T_ values between the test assay and a reference assay with known conidia number (ΔC_T_) and the number of target cell equivalents [75]. In another study, the *Fusarium*-DNA exposure was converted to number of genomes per cubic meters of air (genomes/m^3^) by using the known haploid genome size of *F. graminearum* [99] and the sampled air volume [19].

When choosing exposure denomination it is important to evaluate what is quantified and what is relevant to occupational health. Fungi have many and various forms that may have variable number nuclei. The term spore equivalents may be misleading if the spores have multiple nuclei. However, the aerosolized unit, single or aggregated spores and hyphal fragments, may be most relevant for inhalation. Since the quantification of DNA includes both spores and hyphae, the DNA- based exposure results will be higher than both cultivation- or microscopy-based results.

## 9. OEL for fungi?

Several countries have adopted 8-hour time weighted average occupational exposure limits (OELs) for organic dust at 5 mg/m^3^ [100] and for grain dust of 4 mg/m^3^ [101]. A major problem of using this permissive dust level for evaluation of work-related health risks is that organic dusts consist of a complex mixture of diverse biologically active components which may have additive or synergistic effects. Nevertheless, for fungal spores the combined evidence from human challenge and epidemiological studies support fairly consistent lowest observed respiratory effects levels of approximately 10^5^ spores/m^3^ for diverse fungal species in non-sensitized populations [102]. However, toxigenic fungi are likely to have much lower effect levels, and may also cause other health effects than non-mycotoxin-producing fungi. Species identification, e.g. by PCR, is therefore needed before one can evaluate such data. To confirm exposure, mycotoxins or mycotoxin metabolites may be detected in biological samples [103–105]. Furthermore, biological effect markers of mycotoxin exposure, such as aflatoxin B_1_-N7-guanine adducts, are possible to detect. As intermediate outcomes in the process leading to adverse health effects, such markers are important to evaluate in exposure-response association studies.

## 10. Conclusions

The specificity and sensitivity of the PCR technology makes identification of microorganisms much easier and should therefore be used more in occupational exposure assessments. The detection of DNA sequences related to mycotoxin synthesis indicates presence of fungi with mycotoxin producing potential, and may predict mycotoxin exposure. However, fungal DNA as indicators of trichothecenes presence should be used with caution, as the fungal DNA not necessarily reflects mycotoxins presence.

## 11. Future prospects

The more we learn about non-infectious microorganisms and their effect on human health, the more important becomes species identification. Since various species have different pathogenic potential, species identification is very relevant to health risk assessments. DNA-based detection of specific microbial species or genes related to their toxicity may lead to improved exposure estimates of known microorganisms, and may subsequently provide the possibility to establish OELs for specific fungi and other microorganisms.

An attractive possibility for the future would be the microbial screening of various occupational environments by the microarray technology. Initially, this could be implemented for research purposes, but it could also be a method to identify characteristic microbial profiles of the various occupational environments that subsequently could ease the control measures by rapid screening.
